# PEDF mediates pathological neovascularization by regulating macrophage recruitment and polarization in the mouse model of oxygen-induced retinopathy

**DOI:** 10.1038/srep42846

**Published:** 2017-02-17

**Authors:** Sha Gao, Changwei Li, Yanji Zhu, Yanuo Wang, Ailing Sui, Yisheng Zhong, Bing Xie, Xi Shen

**Affiliations:** 1 Department of Ophthalmology, Ruijin Hospital, Shanghai Jiaotong University School of Medicine, 197 Ruijin 2nd Road, Shanghai, 200025, People’s Republic of China; 2 Shanghai Key Laboratory for Prevention and Treatment of Bone and Joint Diseases with Integrated Chinese-Western Medicine, Shanghai Institute of Traumatology and Orthopedics, Ruijin Hospital, Shanghai Jiaotong University School of Medicine, 197 Ruijin 2nd Road, Shanghai, 200025, People’s Republic of China

## Abstract

Macrophages have been demonstrated to play a proangiogenic role in retinal pathological vascular growth. Pigment epithelium-derived factor (PEDF) works as a powerful endogenous angiogenesis inhibitor, but its role in macrophage recruitment and polarization is largely unknown. To explore the underlying mechanisms, we first evaluated macrophage polarization in the retinas of the oxygen-induced retinopathy (OIR) mouse model. Compared to that in normal controls, M1- and M2-like macrophages were all abundantly increased in the retinas of OIR mice. In addition, both M1 and M2 subtypes significantly promoted neovascularization *in vitro* and *in vivo*. In addition, we found that PEDF inhibited retinal neovascularization by dampening macrophage recruitment and polarization. Furthermore, PEDF inhibited macrophage polarization through adipose triglyceride lipase (ATGL) by regulating the activation of MAPKs and the Notch1 pathway, as we found that the phosphorylation of MAPKs, including p38MAPK, JNK and ERK, as well as the accumulation of Notch1 were essential for hypoxia-induced macrophage polarization, while PEDF significantly dampened M1 subtype-related iNOS and M2 subtype-related Arg-1 expression by inhibiting hypoxia-induced activation of Notch1 and MAPKs through ATGL. These findings reveal a protective role of PEDF against retinal neovascularization by regulating macrophage recruitment and polarization.

Retinopathy of prematurity (ROP) is a common and prevalent blinding disease in infants worldwide^1^,^2^. The pathophysiological course of ROP involves two phases. The first phase begins with delayed retinal vascular growth after birth and partial regression of existing vessels, followed by a second phase of hypoxia-induced pathological vessel growth. Both oxygen-regulated and non-oxygen-regulated factors contribute to normal vascular development and retinal neovascularization^1^,^2^,^3^,^4^. These vascular abnormalities of the retina further damage the retinal microenvironment, thereby establishing a vicious cycle that reinforces ischemia and eventually results in retinal hemorrhage, detachment, fibrosis, and ultimately blindness. Although laser photocoagulation or cryotherapy of the retina reduces the incidence of blindness by 25%, the visual outcomes after treatment are often poor^2^. Understanding the molecular basis of this disease is crucial, as preventive and less destructive therapies are sorely needed.

Recent studies have demonstrated that retinal neovascularization is regulated by a complex interaction between immune cells and inflammatory cytokines. In this process, macrophages are recruited to the hypoxic areas to form a proangiogenic microenvironment^5^,^6^,^7^,^8^. In addition, retinal avascular areas and neovascularization tufts are greatly reduced by general macrophage depletion by clodronate liposomes in OIR mouse model^9^. Plasticity and diversity are fundamental characteristics of macrophages; undifferentiated M0 macrophages can polarize into classical proinflammatory M1-like and alternative anti-inflammatory M2-like macrophages in responses to tissue signals^10^. The M1-like phenotype expresses CCL3, CCL5, CD80 and inducible nitric oxide synthase (iNOS) in responses to the induction of lipopolysaccharide (LPS), tumor necrosis factor-α (TNF-α), interleukin-1β (IL-1β) and IL-23. In contrast, the M2 phenotype expresses CD206, CD163, CCL22 and arginase-1 (Arg-1) under IL-4, IL-10, TGF-β and IL-13 stimulation^10^,^11^. Both M1- and M2-like phenotypes are found in ROP^5^; however, their respective roles in neovascularization are not well studied. Some studies have shown that M2-like macrophages, rather than M1, play a major role in enhancing retinal pathological neovascularization^12^. In contrast, others have reported that Interleukin-17A neutralization alleviates ocular neovascularization by promoting M2 and mitigating M1 macrophage polarization^13^. Therefore, the respective roles of M1- and M2-like macrophages in neovascularization still require further investigation.

Pigment epithelium-derived factor (PEDF) is a 50-kDa protein secreted by the retinal pigment epithelium (RPE) and a select number of other cell types in the eye, as well as other tissues in the body, which protects retinal neurons from light damage, oxidative stress and glutamate excitotoxicity^14^. As a potent antiangiogenic factor, PEDF has been reported to inhibit retinal neovascularization by suppressing vascular endothelial growth factor (VEGF)-induced retinal microvascular endothelial cell proliferation^15^. In addition, PEDF inhibits angiogenesis by directly interfering with the effects of VEGF by enhancing γ-secretase-dependent cleavage of the VEGF receptor-1, which consequently inhibits VEGF-induced angiogenesis^16^,^17^. However, whether PEDF could mediate neovascularization of the retina by regulating macrophage recruitment and polarization in the pathogenesis of ROP still needs to be investigated.

Given that macrophages play a proangiogenic role in retinal pathological vascular growth and PEDF works as a powerful endogenous angiogenesis inhibitor whose role in macrophage recruitment and polarization is largely unknown, we investigated whether PEDF could mediate neovascularization by regulating macrophage recruitment and polarization. Our findings confirmed the respective roles of M1- and M2-like macrophages in the promotion of neovascularization and uncovered a vital role of PEDF in regulating M1- and M2-like macrophage recruitment and polarization. Moreover, PEDF dampened neovascularization by regulating hypoxia-induced MAPKs and Notch1 activation through its receptor ATGL.

## Results

### Both M1- and M2-like macrophages promoted neovascularization

**Figure 1:**
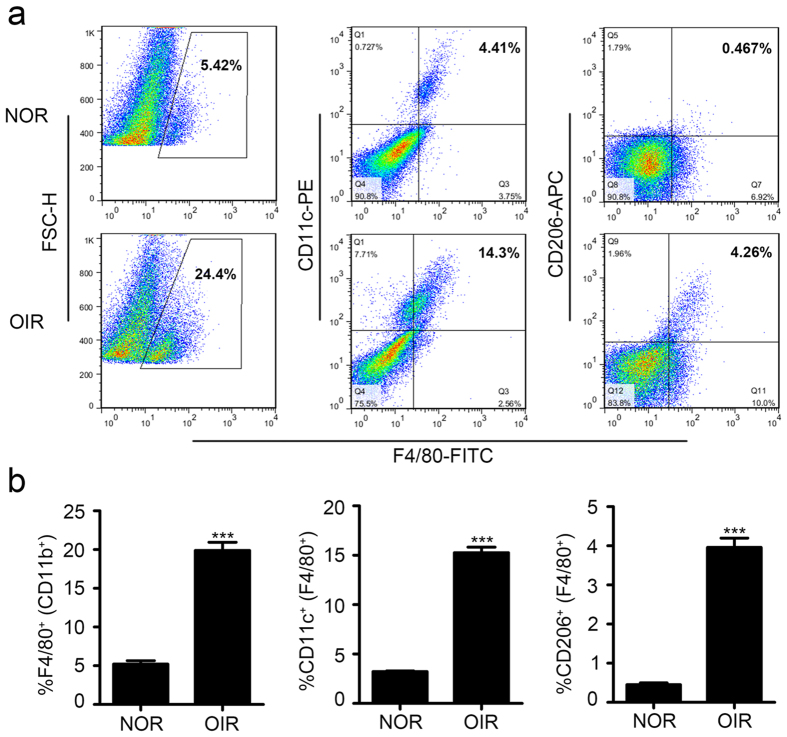
Both M1- and M2-like macrophages infiltrated in the retinas of OIR mouse model. (**a,b**) Flow cytometry analysis of macrophages infiltration (F4/80^+^), M1-like macrophages (F4/80^+^ and CD11C^+^) and M2-like macrophages (F4/80^+^ and CD206^+^). ***P < 0.001. *P* values were analyzed by t-test. All data are representative of three independent experiments with n = 7–8 per group.

To better understand which phenotype of macrophage (M1 or M2) plays a vital role in the pathologic neovascularization in ROP, we first evaluated the infiltration of M1- and M2-like macrophages in the oxygen-induced retinal (OIR) neovascularization mouse model. The flow cytometry results showed that in addition to M1 macrophages, there were many more M2 macrophages that had infiltrated in the retinas of the OIR model than that of the control group, and the M2 macrophages accounted for approximately 15% and 4% of the total macrophages, respectively (Fig. 1a,b). This result revealed that in the progress of neovascularization, both M1 and M2 macrophages were infiltrated in the retinas of the OIR mouse model.

**Figure 2:**
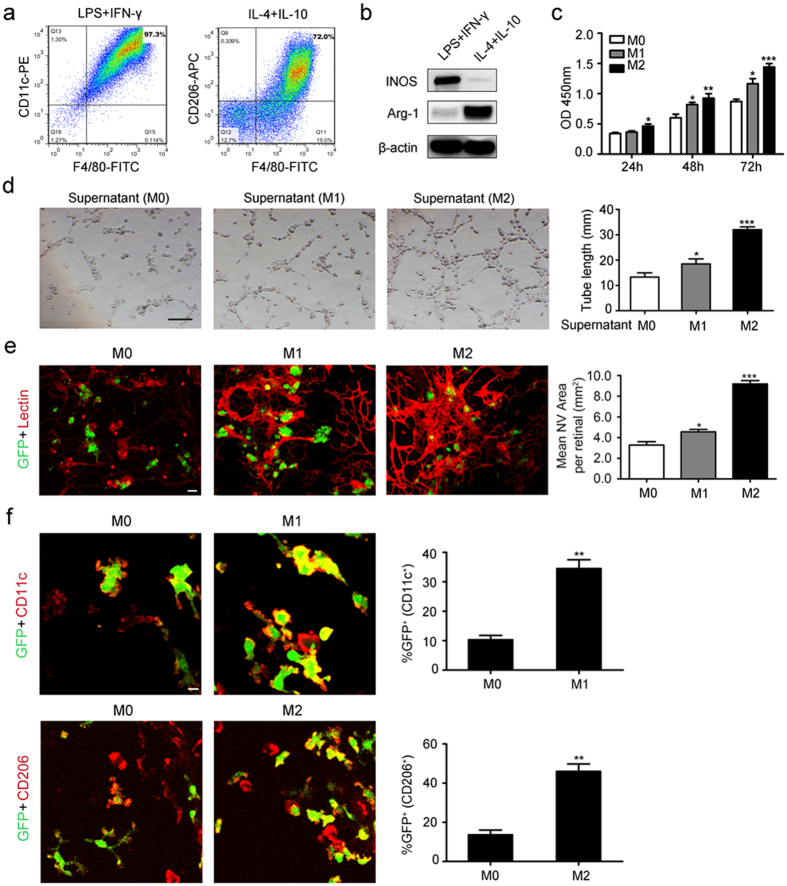
Both M1- and M2-like macrophages promoted pathologic neovascularization. (**a**) Flow cytometry analysis of M1-like macrophages infiltration (F4/80^+^ and CD11C^+^) induced by LPS plus IFN-γ and M2-like macrophages infiltration (F4/80^+^ and CD206^+^) induced by IL-4 plus IL-10. (**b**) Western blot analysis of iNOS and Arg-1 post 24 hours of LPS plus IFN-γ or IL-4 plus IL-10 stimulation. (**c**) Time-curve of HUVECs proliferation induced by M0, M1 or M2 macrophages. (**d**) HUVECs tube formation post 24 hours of M0, M1 or M2 macrophages stimulation. Scale bar represents 100 μm. (**e**) Immunofluorescence analysis of lectin in the retinas of OIR mice after 5 days of EGFP-labeled M0, M1 or M2 macrophages intravitreal injection. Scale bars represent 10 μm. (**f**) Immunofluorescence analysis of CD11C^+^ and CD206^+^ macrophages in the retinas of OIR mice after 5 days of EGFP-labeled M0, M1 or M2 macrophages intravitreal injection. Scale bars represent 10 μm. *P < 0.05, **P < 0.01, ***P < 0.001. *P* values were analyzed by one-way ANOVA in (**c–e**) and t-test in (**f**). All data are representative of three independent experiments and are means ± SEM.

We next sought to explore the function of these two subtypes in neovascularization. First, we polarized M0 macrophages into M1- or M2-like macrophages by LPS plus IFN-γ or IL-4 plus IL-10, respectively. The flow cytometry results showed that there were approximately 97.3% F4/80^+^ and CD11c^+^ macrophages after 24 hours of incubation with LPS plus IFN-γ, while F4/80^+^ and CD206^+^ macrophages accounted for 72% of the total after 24 hours of incubation with IL-4 plus IL-10 (Fig. 2a). Furthermore, the expression of iNOS and Arg-1 detected by western blot, immunofluorescence analysis or quantitative real time RT-PCR also confirmed that LPS plus IFN-γ or IL-4 plus IL-10 successfully polarized M0 macrophages into M1- or M2-like macrophages, as LPS plus IFN-γ significantly induced iNOS expression, while IL-4 plus IL-10 abundantly increased Arg-1 expression (Fig. 2b and Supplementary Figure 1).

To detect the function of M1- and M2-like macrophages in the neovascularization process, we incubated human umbilical vein endothelial cells (HUVECs) together with the supernatant of M1- or M2-like macrophages. The proliferation rate showed that HUVECs grow much faster incubated with the supernatant of M1- or M2-like macrophages than M0-like macrophages. We also found that M2-like macrophages had a much greater potential to promote HUVEC proliferation, as a significant difference in the cell number began to arise after 48 hours of incubation with the supernatant of M1 macrophages compared with that of M0 macrophages, while the significant difference arose after only 24 hours of incubation with the supernatant of the M2 macrophages (Fig. 2c). Furthermore, we also found that even though M1 macrophages had less of an ability to promote tube formation than M2 macrophages, there was much more tube formation in HUVECs after 24 hours of incubation with M1 or M2 macrophages than M0 macrophages (Fig. 2d). In addition, the lectin immunofluorescence analysis showed that intravitreal injection of both EGFP-labeled M1- and M2-like macrophages abundantly increased the retinal pathologic angiogenesis area (Fig. 2e). The results also showed that after 5 days of injection, the transplanted M1 (CD11C^+^) and M2 (CD206^+^) macrophages were detected around the preretinal neovascular tufts, about 40% of M1 and 50% of M2 macrophage retained the polority (Fig. 2f). Taken together, all of these data demonstrated that both M1 and M2 macrophages had the potential ability to promote retinal pathologic neovascularization.

### PEDF dampened pathologic neovascularization in the retinas of OIR mice by inhibiting macrophage recruitment

**Figure 3:**
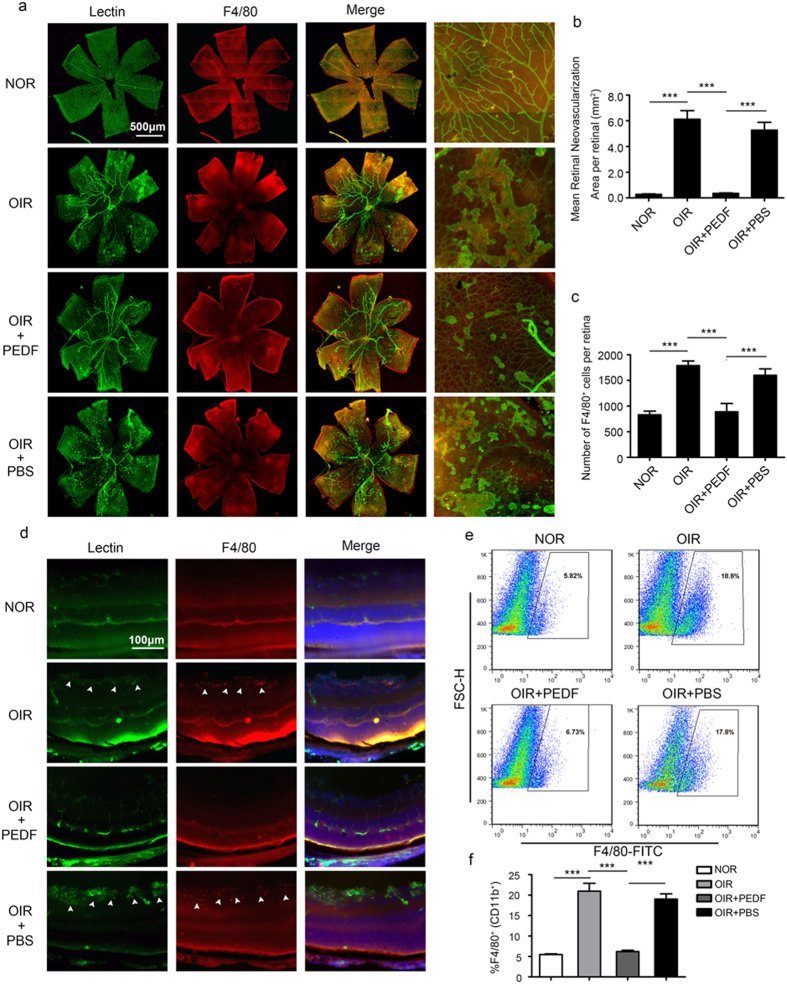
PEDF dampened macrophages infiltration and neovascularization in retinas of OIR mice. (**a–c**) Immunofluorescence analysis of lectin and F4/80 recruitment in the retinal flat-mounts of normal and OIR mouse at postnatal day 17 treated with or without PEDF. Rightmost designate region of 200x magnification. (**b,c**) Local neovascularization (**b**) and macrophages infiltration (**c**) in retinas of normal and OIR mice at postnatal day 17 treated with or without PEDF. (**d**) Immunofluorescent staining of lectin and F4/80 in the retinas of normal and OIR mice at postnatal day 17 treated with or without PEDF. The arrow designates region of neovascularization and macrophage infiltration. (**e,f**) Flow cytometry analysis of macrophages (F4/80^+^) infiltration in the retinal of normal and OIR mice at postnatal day 17 treated with PEDF or not. ***P < 0.001. *P* values were analyzed by one-way ANOVA. All data are representative of three independent experiments with n = 6 per group and are means ± SEM.

Although PEDF is known to have a potent antiangiogenic activity^18^, few reports have shown the function of PEDF in neovascularization of OIR. To explore the role of PEDF in mediating pathologic neovascularization of OIR, we detected neovascularization in the retinas of the OIR mouse model treated with or without PEDF. Compared to that of the normal controls, the lectin immunofluorescence analysis of retinal flat-mounts showed that oxygen induced a significant increase in the total pathologic neovascularization area, but this process was significantly dampened by PEDF; the high magnification of retinal flat-mounts showed fewer and smaller neovascularization sprouts in the PEDF-treated group than in controls (Fig. 3a,d). PEDF also significantly inhibited oxygen-induced macrophage recruitment in the retinas as shown by F4/80 immunofluorescence staining (Fig. 3a,d). In addition, PEDF-inhibited neovascularization and macrophage recruitment were also confirmed by lectin and F4/80 immunofluorescence analysis in the extended retinas (Fig. 3b,c). Finally, the flow cytometry analysis of F4/80^+^ myeloid cells further confirmed that PEDF inhibited macrophage recruitment (Fig. 3e,f). Taken together, these data demonstrated that PEDF dampened neovascularization by inhibiting macrophage recruitment.

### PEDF dampened neovascularization by mediating macrophage polarization in OIR

**Figure 4:**
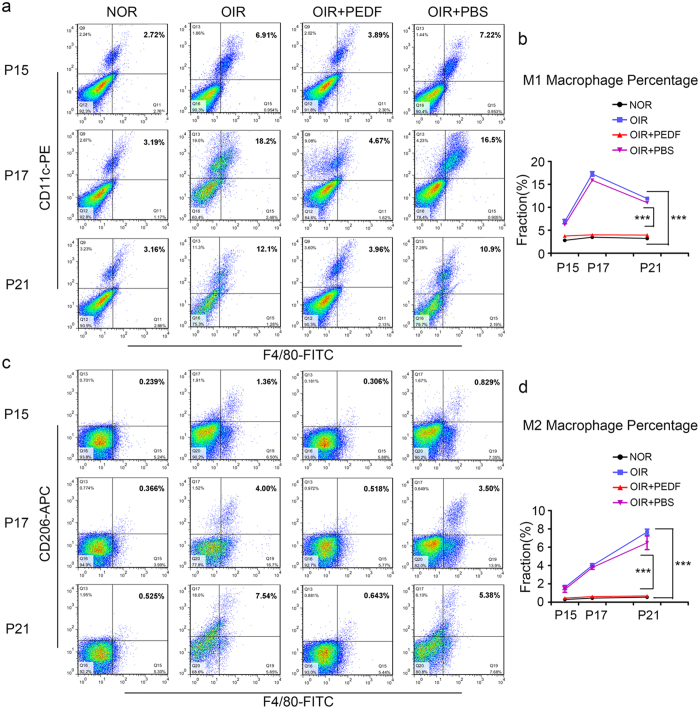
Time curve of M1-like and M2-like macrophages infiltration in the retinas of normal and OIR mice treated with or without PEDF. (**a–d**) Flow cytometry analysis of M1-like macrophages (F4/80^+^ and CD11C^+^) (**a,b**) and M2-like macrophages (F4/80^+^ and CD206^+^) (**c,d**) infiltration in the retinal of normal and OIR mice at postnatal day 15, 17 and 21 treated with or without PEDF. ***P < 0.001. P values were analyzed by two-way ANOVA. All data are representative of three independent experiments with n = 5 per group and are means ± SEM.

**Figure 5:**
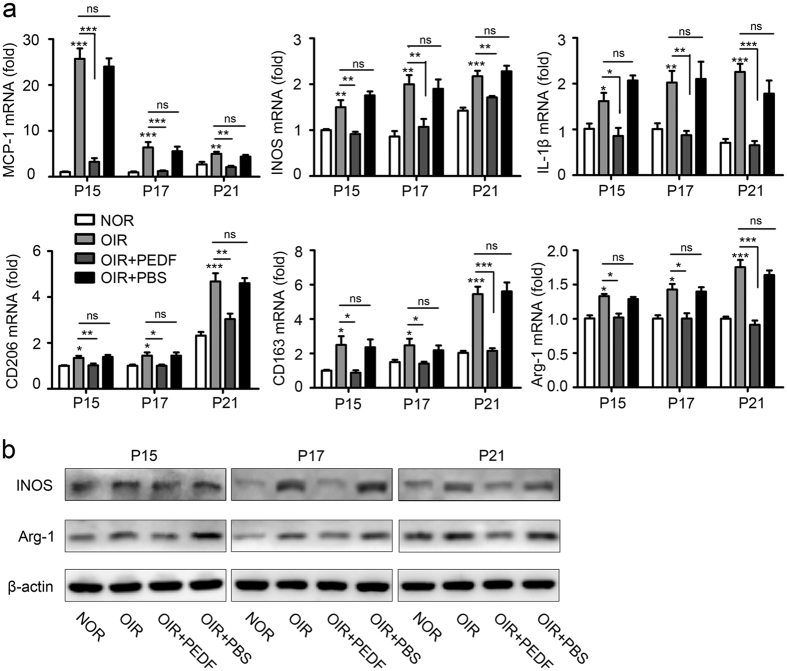
PEDF dampened neovascularization by mediating macrophage polarization in OIR mice. (**a**) Quantification of M1-like macrophages related MCP-1, iNOS, IL-1β and M2-like macrophages related CD206, CD163, Arg-1 mRNA expression in the retinal of normal and OIR mice at postnatal day 15, 17 and 21 treated with or without PEDF. (**b**) Western blot analysis of iNOS and Arg-1 expression in the retinal of normal and OIR mice at postnatal day 15, 17 and 21 treated with PEDF or not. *P < 0.05, **P < 0.01, ***P < 0.001. P values were analyzed by one-way ANOVA. All data are representative of three independent experiments with n = 5 per group and are means ± SEM.

Having observed that PEDF dampened neovascularization by inhibiting macrophage recruitment while our results in Fig. 2 also confirmed that both M1- and M2-like macrophages promoted neovascularization, we hypothesized that PEDF might mediate neovascularization by regulating macrophage polarization. To test our hypothesis, we first detected M1 and M2 macrophage polarization in OIR. The flow cytometry results showed that PEDF significantly reduced oxygen-induced M1 (Fig. 4a,b) and M2 subtype (Fig. 4c,d) polarization in retinas. The time curve of the quantitative mRNA expression results also showed that PEDF not only significantly inhibited oxygen-induced M1 macrophage activation-related MCP-1, iNOS and IL-1β expression but also dampened M2 macrophage activation-related CD206, CD163 and Arg-1 expression (Fig. 5a). This process was further confirmed by the iNOS and Arg-1 expression detected by western blot (Fig. 5b). Taken together, all of these data demonstrated that PEDF dampened neovascularization by mediating macrophage polarization in OIR.

**Figure 6:**
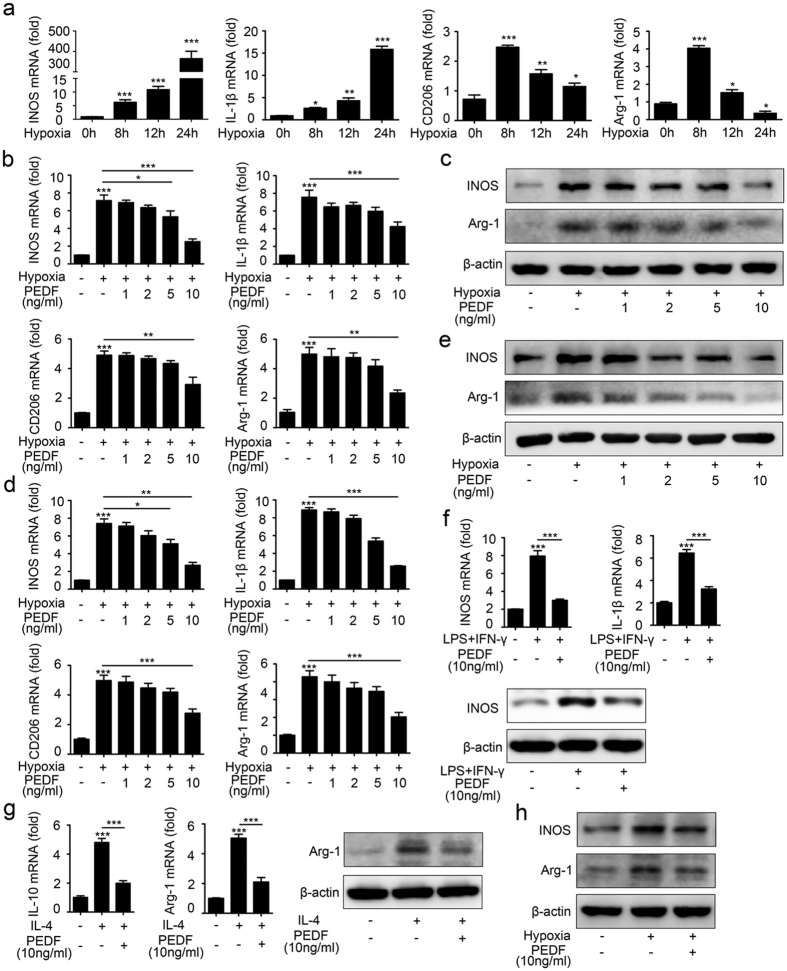
PEDF mediated hypoxia-induced macrophage polarization. (**a**) Time-curve expression of M1-like macrophage related iNOS, IL-1β and M2-like macrophage related CD206, Arg-1 expression in BMDMs induced by hypoxia. (**b**) Quantification of iNOS, IL-1β, CD206 and Arg-1 in BMDMs induced by hypoxia in the presence of different doses of PEDF. (**c**) Western blot analysis of iNOS and Arg-1 in BMDMs induced by hypoxia in the presence of different doses of PEDF. (**d**) Quantification of iNOS, IL-1β, CD206 and Arg-1 in RAW264.7 cells induced by hypoxia in the presence of different doses of PEDF. (**e**) Western blot analysis of iNOS and Arg-1 in RAW264.7 cells induced by hypoxia in the presence of different doses of PEDF. (**f**) Quantification of iNOS and IL-1β and western blot analysis of iNOS expression in MDMs induced by LPS plus IFN-γ. (**g**) Quantification of IL-10 and Arg-1 and western blot analysis of Arg-1 expression in MDMs induced by IL-4. (**h**) Western blot analysis of iNOS and Arg-1 expression in MDMs induced by hypoxia. *P < 0.05, **P < 0.01, ***P < 0.001. P values were analyzed by two-way ANOVA. All data are representative of three independent experiments and are means ± SEM.

We next sought to detect whether PEDF could mediate macrophage polarization directly *in vitro*. To mimic the hypoxia-induced OIR model, we cultured macrophages in 1% oxygen. The time curve of mRNA expression showed that M1 macrophage-related iNOS and IL-1β expression was elevated within 8 hours and increased up to 24 hours after hypoxia, while increased M2 macrophage-related CD206 and Arg-1 expression peaked at 8 hours (Fig. 6a). These results indicated that M2 macrophages might play a role in the beginning process of neovascularization, and M1 macrophages might function in the later process of neovascularization. Next, we detected whether PEDF could inhibit hypoxia-induced macrophage polarization. The quantitative mRNA expression levels of iNOS, IL-1β, CD206 and Arg-1 in primary bone marrow-derived macrophages (Fig. 6b) and in RAW264.7 cells (Fig. 6d) all showed that PEDF dampened both M1 and M2 macrophage polarization. In addition, we found that the expression of these hypoxia-induced macrophage activation-related genes was decreased by PEDF at a concentration of 10 ng/ml. Finally, the protein expression of iNOS and Arg-1 in primary bone marrow-derived macrophages (Fig. 6c) and RAW264.7 cells (Fig. 6e) further confirmed this process. We next sought to detect whether the polarizing effect of PEDF on macrophages was also seen in human primary macrophages. Human monocytes purified from peripheral blood mononuclear cells (PBMCs) of 4 healthy donors were treated with or without IFN-γ plus LPS or IL-4 alone to induce their polarization into M1 or M2 cells, respectively, according to a published protocol optimized for human monocyte-derived macrophages (MDMs)^19^,^20^. The results showed that in addition to inhibiting M1 macrophage-related IL-1β and iNOS expression induced by IFN-γ plus LPS (Fig. 6f), PEDF preincubation also dampened M2 macrophage-associated IL-10 and Arg-1 expression induced by IL-4 (Fig. 6g). In addition, the western blot results also revealed that 10 ng/ml PEDF significantly dampened hypoxia-upregulated iNOS and Arg-1 expression in human MDMs (Fig. 6h). Together, all of these data demonstrated that PEDF dampened macrophage polarization induced by hypoxia *in vitro*.

### PEDF mediated macrophage polarization through ATGL

**Figure 7:**
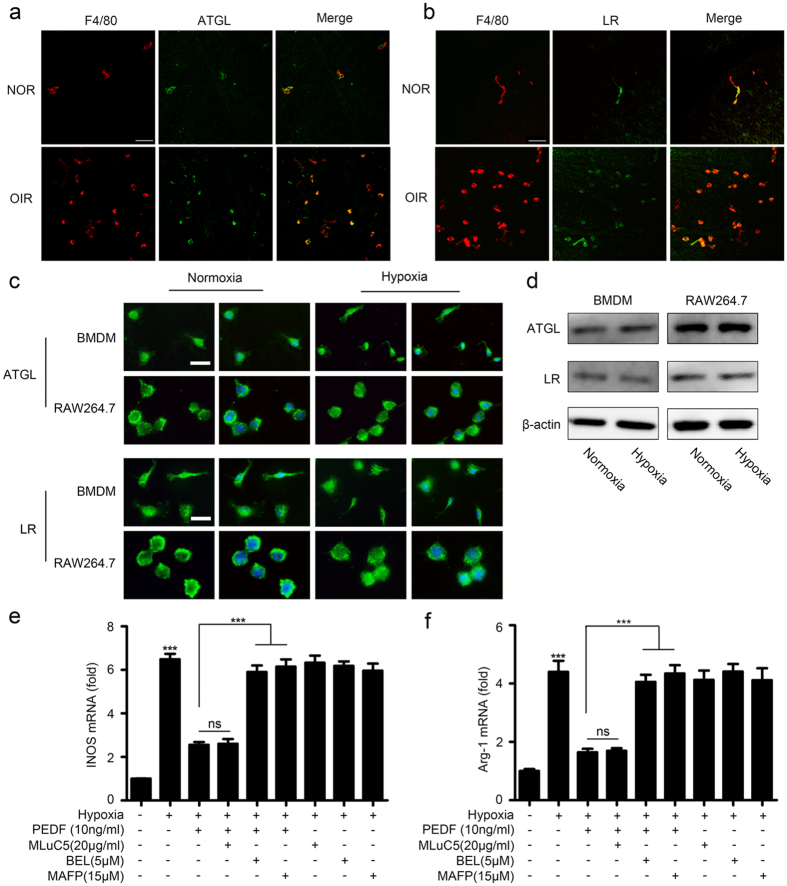
PEDF regulated hypoxia-induced macrophages polorization via ATGL. (**a,b**) Immunofluorescence analysis of ATGL and LR expression on macrophages of retinas in the OIR model by ATGL plus F4/80 (**a**) or LR plus F4/80 (**b**) staining. Scale bars represent 50 μm. (**c**) Immunofluorescence analysis of ATGL and LR expression in macrophage RAW264.7 and BMDMs with or without hypoxia treatment. Scale bars represent 10 μm. (**d**) Western blot analysis of ATGL and LR expression in macrophages RAW264.7 and BMDMs with or without hypoxia treatment. (**e,f**) Quantification of iNOS and Arg-1 expression in BMDMs induced by hypoxia prior to stimulation with PEDF and/or ATGL specific inhibitor BEL/ATGL selective inhibitor MAFP/LR antibody MluC5. ***P < 0.001, P values were analyzed by one-way ANOVA. All data are representative of three independent experiments and are means ± SEM.

PEDF has been reported to bind specifically to two known cell membrane receptors, adipose triglyceride lipase (ATGL)/independent phopholipase A2 (iPLA2) in neuronal cells^21^ and laminin receptor (LR) in endothelial cells^22^. Some studies have also revealed that PEDF induces THP-1 macrophage apoptosis and may exert an anti-inflammatory property by increasing IL-10 expression in macrophages^23^,^24^. Moreover, ATGL and LR are all expressed by macrophages, and ATGL is essential for PEDF-induced macrophage activation in obesity-associated inflammation^25^. Therefore, we hypothesized that PEDF-mediated macrophage polarization in response to hypoxia might be via ATGL and/or LR. In order to test our hypothesis, we first detected ATGL and LR expression in macrophages of retinas of OIR mice. The double-staining for F4/80 plus ATGL and F4/80 plus LR revealed that both ATGL and LR were expressed on macrophages of the retinas in the normal and the OIR mice (Fig. 7a,b). Furthermore, theimmunofluorescence and western blot analysis results showed that ATGL and LR were constitutively expressed in both RAW264.7 macrophages and primary bone marrow-derived macrophages (BMDMs) (Fig. 7c,d). Next, we investigated whether phospholipase enzymatic activity of the ATGL receptor was required for PEDF-induced inflammatory responses. Macrophages were incubated with the specific iPLA2 inhibitor, bromoenol lactone (BEL)^25^ (Cat No: B1552, Sigma, USA) prior to stimulation with PEDF. Inhibition of phospholipase activity significantly dampened PEDF-inhibited M1 macrophage-related iNOS and M2 macrophage-related Arg-1 expression (Fig. 7e,f). Furthermore, the requirement for phospholipase activity of ATGL for PEDF-mediated macrophages polarization was further confirmed by culturing macrophages with an iPLA2/ATGL selective inhibitor, methyl arachidonyl fluorophosphonate (MAFP)^26^ (Cat No: 70660, Cayman Chemical, USA) prior to the addition of PEDF. MAFP attenuated PEDF-inhibited macrophage polarization-associated gene expression (Fig. 7e,f). These results suggest that Ca2^+^ -independent phospholipase activity is involved in PEDF-mediated macrophage polarization in response to hypoxia. Next, we explored whether LR was involved in the effects of PEDF on macrophage polarization. We stimulated macrophages with recombinant PEDF in the presence or absence of an anti-LR antibody, MLuC5^27^ (Cat No: ab3099, Abcam, USA). As shown in Fig. 7c,d, inhibition of the PEDF–LR interaction with the LR-specific antibody did not affect PEDF-inhibited iNOS and Arg-1 expression. Together, these results indicated that ATGL, rather than LR, was required for PEDF-mediated macrophage polarization, although ATGL and LR were both constitutively expressed in macrophages.

### PEDF mediated macrophage polarization by regulating MAPKs and Notch 1 activation

**Figure 8:**
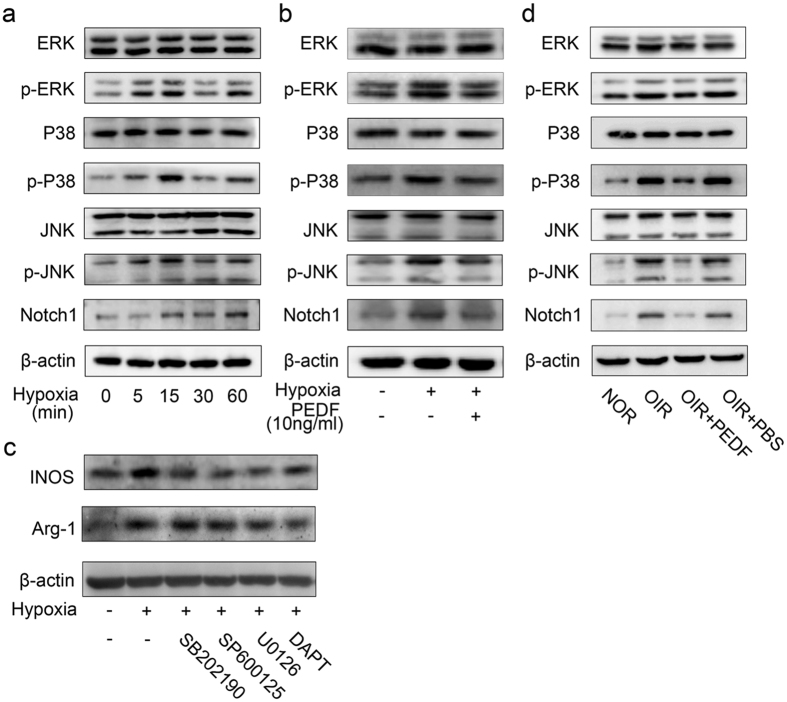
PEDF mediated macrophage polarization via MAPKs and Notch1 pathway. (**a**) Time curve of MAPKs, including p38MAPK, JNK and ERK phosphorylation and Notch1 accumulation induced by hypoxia in BMDMs. (**b**) Phosphorylation of p38MAPK, JNK, ERK and accumulation of Notch1 in BMDMs induced by hypoxia pretreated with or without PEDF. (**c**) Quantitation of iNOS and Arg-1 expression in BMDMs induced by hypoxia pretreated with p38MAPK inhibitor SB202190, JNK inhibitor SP600125, ERK inhibitor U0126 and Notch1 inhibitor DAPT(GSI-IX). (**d**) Phosphorylation of p38MAPK, JNK, ERK and accumulation of Notch1 in the macrophages isolated from the retinal of normal and OIR mice treated with or without PEDF. All data are representative of three independent experiments with n = 5 per group and are means ± SEM.

Having observed that PEDF dampened hypoxia-induced macrophage polarization via ATGL, we next sought to explore the key pathways downstream of ATGL through which PEDF regulates hypoxia-induced macrophage polarization. First, we found that hypoxia induced MAPK phosphorylation, including phosphorylation of p38MAPK, JNK and ERK, as well as Notch1 accumulation in a time-dependent manner (Fig. 8a), whereas these processes were abundantly dampened by PEDF pre-incubation in BMDMs (Fig. 8b). Furthermore, the p38MAPK inhibitor SB202190, JNK inhibitor SP600125, ERK inhibitor U0126 and Notch1 pathway inhibitor DAPT(GSI-IX) significantly dampened hypoxia-induced M1 macrophage activation-related iNOS and M2 macrophage activation-related Arg-1 expression (Fig. 8c). To further demonstrate that PEDF mediated macrophage polarization by regulating activation of MAPKs and the Notch pathway, we next sought to explore whether PEDF could regulate the activation of MAPKs and the Notch pathway in the OIR model. The results also showed that MAPK phosphorylation and Notch1 accumulation were abundantly increased in the macrophages isolated from retinas of the OIR mice; however, the increase was significantly dampened by PEDF treatment (Fig. 8d). Taken together, all of these data demonstrated that PEDF mediated macrophage polarization by regulating the activation of MAPKs and the Notch1 pathway.

## Discussion

Vascular regression, aberrant angiogenesis, vascular leakage, and inflammatory cell infiltration are the main underlying causes for the exacerbation of major retinal vascular diseases such as proliferative diabetic retinopathy (PDR) and retinopathy of prematurity (ROP)^28^. Previous studies have demonstrated that macrophages play a proangiogenic role in retinal pathological vascular growth, and both M1- and M2-like subtypes are found in ROP^5^; however, their respective roles in neovascularization are controversial. Here, we observed that both M1- and M2-like macrophages increase in response to the ischemic condition, and most of these macrophages were closely related to the neovascularization tufts on the inner surface of the retinas in OIR. Furthermore, both M1- and M2-like subtypes promoted HUVEC proliferation and tube formation as well as significantly increased retinal pathologic neovascularization. PEDF works as a powerful endogenous angiogenesis inhibitor, but its role in macrophage recruitment and polarization is largely unknown. Our results revealed that in addition to dampening macrophage recruitment, PEDF also inhibited macrophage polarization to mediate retinal neovascularization. Furthermore, PEDF mediated macrophage polarization by regulating phosphorylation of MAPKs, including phosphorylation of p38MAPK, JNK and ERK, as well as Notch1 accumulation through its receptor ATGL. Therefore, the identification of the promotion of retinal neovascularization by M1- and M2-like macrophages and the elucidation of the function of PEDF in mediating macrophage recruitment and polarization provides a new mechanism for the regulation of retinal neovascularization by PEDF.

Plasticity and diversity are fundamental characteristics of macrophages. Undifferentiated M0 macrophages can polarize into classical proinflammatory M1-like and alternative anti-inflammatory M2-like macrophages in responses to the tissue microenvironment^10^, but, actually, macrophages usually do not form a stable phenotype in response to a combination of factors present in the tissue^20^,^29^. Our data and previous reports have demonstrated both M1- and M2-like macrophages in ROP and an OIR mouse model^5^; however, their respective roles in neovascularization are controversial. Here, we found that although the M1 phenotype had less of a proangiogenesis ability than M2-like macrophages, both M1- and M2-like macrophages significantly promoted HUVEC proliferation and tube formation *in vitro*. In addition, intravitreal injection of M1- and M2-like macrophages abundantly increased the retinal pathologic neovascularization area. These data demonstrated that both M1- and M2-like macrophages promoted the retinal pathologic neovascularization process.

Although the intrinsic mechanisms by which macrophages, including the M1- and M2-like subtype, are involved in the neovascularization process are not fully understand, some studies have proven that VEGF from M1-like macrophages is essential for ocular neovascularization^13^. Other research suggests that M2 macrophages secrete endothelial cell growth and proangiogenic factors such as VEGF, CXCL1 and IL-19, which are considered to promote wound repair and neovascularization^30^,^31^,^32^. Consistent with these findings, our results also confirmed that both M1- and M2-like macrophages secreted more VEGF than M0 macrophages (data not shown). Furthermore, we also found that both M1- and M2-like macrophages increased VEGFR expression in HUVECs compared with M0 macrophages (Supplementary Figure 2).

Angiogenesis, defined as the process of the formation of new capillaries from pre-existing vessels, is a complex multistep process. Various angiogenic stimulators and inhibitors regulate this complex process that includes activation, migration and proliferation of endothelial cells, the remodeling of the extracellular matrix, the formation of a lumen and the functional maturation of the vessels^16^,^33^. PEDF is actually one of the strongest natural inhibitors of pathological angiogenesis known to date and is predicted to be a promising therapeutic tool for slowing the progression of many neovascular pathologies, as it is endogenously present in the body, would not be expected to activate drug-resistant genes, and meets the criteria, as it specifically and potently suppresses pathogenic neovessel growth without harming mature vessels^34^,^35^. PEDF-deficient mice exhibit an enhanced rate of retinal vascular expansion and are more sensitive to hyperoxia-mediated vessel obliteration^36^. In addition, systemic delivery of recombinant PEDF prevents ischemia-induced retinopathy^37^. Furthermore, it has been demonstrated that penetrating ocular injury increases PEDF expression in the retina^38^,^39^, and endogenous PEDF has an antiangiogenic function in the OIR model^39^. However, little is known regarding whether PEDF can mediate retinal neovascularization by regulating macrophage recruitment and polarization in OIR. Here, we found that in addition to inhibiting macrophage infiltration, PEDF also dampened macrophage polarization in the mouse model of OIR. Furthermore, PEDF mediated macrophage polarization by regulating the phosphorylation of MAPKs, including p38MAPK, JNK, ERK, and Notch1 accumulation through its receptor ATGL.

The multifunctionality of PEDF could be explained by the interactions with different cell surface receptors of target cells^40^. Studies have revealed that PEDF induces THP-1 macrophage apoptosis and may exert anti-inflammatory properties by increasing IL-10 expression in macrophages^23^,^24^. Moreover, ATGL and LR, which have been identified as receptors of PEDF in neuronal^21^ and endothelial cells^22^, respectively, are both expressed by macrophages, and ATGL is essential for PEDF-induced macrophage activation in obesity-associated inflammation^25^. Consistently, we found that PEDF mediates macrophage polarization also via ATGL, although LR is also detectable in macrophages. Studies have shown that three parallel signal transduction modules of mitogen-activated protein kinases (MAPK), including p38, JNK and ERK, are involved in the promotion of M2 macrophage polarization^13^,^41^. Additionally, the Notch1 receptor plays a pivotal role in M1 macrophage differentiation and heightened inflammatory responses, and inhibition of Notch1 subsequent downstream signaling enhanced M2-polarized macrophage outcomes and promoted anti-inflammatory mediation^42^. Our results showed that in addition to M1- and M2-like macrophage polarization, we also found phosphorylation of MAPKs (including p38, JNK and ERK) and accumulation of Notch1 in macrophages induced by hypoxia and in the retinas of OIR mice. In addition, MAPK inhibitors significantly dampened hypoxia-induced M2-like macrophage-related Arg-1 expression, while Notch1 inhibition abundantly blocked M1-like macrophage-related iNOS expression. These findings provide evidence that activation of MAPKs is crucial for M2-like macrophage polarization, while the Notch1 pathway is essential for M1-like macrophage polarization in OIR mice.

Simultaneously, the increased pathologic angiogenesis by the injection of M1 and M2 macrophages somehow conflicts with a series of studies from Martin Friedlander’s lab. For example, Ritter *et al*. reported that myeloid progenitors transplanted into retina will differentiate into microglia and dramatically enhance revascularization of ischemic areas in the neonatal mouse retina while concomitantly reducing preretinal neovascular tuft formation^8^. In addition, the results from Marchetti *et al*. revealed that umbilical cord blood (UCB)–derived M2 macrophages provide rescue effects in a mouse model of OIR by promoting physiological angiogenesis and reducing associated inflammation^43^. There are two types of retinal neovascularization in the OIR model: one is the pathological neovascularization with a sprouting of abnormal vessels from the surface of the retina into the vitreous, and the other is a physiological revascularization of avascular areas with functional intraretinal vasculature^12^. We speculated that the location of transplanted macrophages in the retina might be responsible for this contradictory effect. After intraocular injection of EGFP-labeled M1 and M2 macrophages, we found that CD11C^+^ and CD206^+^ macrophages were detected around the neovascular tufts at P17 (Fig. 2e). These M1 and M2 macrophages expressed high levels of VEGF, and both M1- and M2-like macrophages increased VEGFR expression in HUVECs compared with M0 macrophages (Supplementary Figure 2); therefore, these macrophages might stimulate endothelial proliferation, thus contributing to the generation of neovascular tufts (NVTs). However, Ritter *et al*. found large numbers of transplanted cells localizing to the central ischemic retina where many endogenous microglia were lost. This localization might replace the function of lost microglia and establish the appropriate gradients of angiogenic factors that promote the controlled revascularization of the damaged areas^8^. In addition to promoting physiological angiogenesis, Marchetti *et al*. also demonstrate that M2 macrophages promote tissue remodeling and repair by reducing inflammatory processes associated with hypoxic damage in the retina^43^. Together, these findings predict that macrophages may have multiple function during angiogenesis depending on differences in the experimental designs. The potential utility of these cells in treating ischemic retinopathies still requires further investigation.

In conclusion, our findings confirm that both M1- and M2-like macrophages are essential for retinal neovascularization and uncover a vital protective role of PEDF against retinal neovascularization by regulating macrophage recruitment and polarization.

## Materials and Methods

### Mice

C57BL/6 mice were purchased from Shanghai Laboratorial Animal Center, Chinese Academy of Sciences (Shanghai, China). 7-weeks old EGFP-labeled (C57BL/6-Tg (CAG-EGFP) C14-Y01-FM131Osb) mice were purchased from Model Animal Research Center of Nanjing University (MARC, Nanjing, China). Mice were housed in the specific pathogen-free animal facility at Ruijin Hospital, Shanghai Jiao Tong University School of Medicine. Mice were maintained in a temperature-controlled (23 °C) facility with a strict 12 hour light/dark cycles and were given free access to food and water.

### Ethics statement

All human sample acquisitions were approved by the ethical committee of Ruijin Hospital, Shanghai Jiao Tong University School of Medicine, China, and performed in accordance with the declaration of Helsinki Principles. All participants provided written informed consent which was obtained before enrolment in the study. All animal experiments were performed according to the protocol approved by Ruijin Hospital, Shanghai Jiao Tong University School of Medicine Animal Care and Use Committee and in direct accordance with Ministry of Science and Technology of the People’s Republic of China on Animal Care guidelines. The protocol was approved by Shanghai Jiao Tong University Animal Care and Use Committee. All surgeries were performed under anesthesia and all efforts were made to minimize suffering.

### Mouse model of oxygen-induced retinopathy (OIR)

Mice on a C57BL/6 background were exposed to 75 ± 3% oxygen from postnatal day 7 (P7) to P12 with their nursing mother and returned to room air at P12 as described in a previous study^44^. Mice were received intravitreal injections of recombinant human PEDF (2 μg/eye, 1 μl; Peprotech, Rocky Hill, NJ) in one eye and PBS in the contralateral eye at P12 and P14. EGFP-labeled M0-, M1- or M2-like macrophages were injected into the vitreous of OIR mice at P12 as reported before^10^. Intraocular injection was performed using a dissecting microscope with a Harvard Pump Microinjection System (Harvard Apparatus, Holliston, MA) and pulled glass micropipettes as described in a previous study^45^. Mice were killed at p15, p17 or p21 and their eyes were removed for immunofluorescent staining or for RNA and protein collection or flow cytometry analysis.

### Isolation of human monocytes and differentiation into MDM

PBMC were isolated from the buffy coats of 4 healthy blood donors by Ficoll-Hypaque density gradient centrifugation. The protocol for isolation and differentiation were according to the previous report^19^.

### Immunofluorescent staining and immunoblot

Mice with oxygen-induced retinopathy or age-matched controls were killed at P17. Their eyes were either enucleated and rapidly frozen in optimum cutting temperature embedding compound (Miles Laboratories) for frozen section (10 μm) or fixed in 4% formalin (2 hours) for flat-mounts analysis. After fixtion, the sections and/or the retinal flat-mounts were incubated with Fluorescein labeled Griffonia Simplicifolia Lectin I (GSL I) isolectin B4 (Cat: FL-1201, Vector Laboratories), DyLight 594 Labeled Griffonia Simplicifolia Lectin I (GSL I) isolectin B4 (Cat: DL-1207, Vector Laboratories), PE-F4/80 (Cat: 12–4801–82, eBioscience) plus Lectin, PE-CD11c (Cat:12-0114-82, eBioscience), PE-CD206 (Cat: 12-2061-80, eBioscience), ATGL (Cat: #2138, Cell Signal Technology) plus F4/80 or LR (Cat: ab133645, Abcam) plus F4/80. After reprobed with Alexa Fluor^®^ 488 conjugated goat anti-Rabbit (Cat: A-11008, Invitrogen), the sections were mounted in ProLong Gold antifade reagent with DAPI (Invitrogen) and captured with a fluorescence microscope (Leica).

The retinal tissues obtained from the OIR and the control mice or cells stimulated as described were lysed using RIPA buffer (PH 7.4) containing protease inhibitor cocktail (Roche). 20 μg of total protein was used for western blot. Arg-1, iNOS, P-p38, P-JNK and P-ERK were detected with Arg-1 antibody (Cell signal technology), iNOS antibody (Abcam), P-p38 antibody (Cell signal technology), P-JNK antibody (Cell signal technology) and P-ERK antibody (Cell signal technology), respectively.

### ATGL and LR inhibition in BMDMs

Primary bone marrow derived macrophages (BMDMs) were cultured in α-MEM (Invitrogen) medium containing 10% FBS (GIBCO), 50 U.ml^−1^ penicillin and 50 ug.ml^−1^ streptomycin (GIBCO) under standard culture conditions. For all cell stimulation experiments, 10^5^ cells were seeded in each well of 24-well plates. When cells were grown to 80% confluence, the indicated doses of ATGL inhibitor (bromoenol lactone (BEL)^25^, final concentration: 5 μM, Cat: B1552, Sigma, USA; methy arachidonyl fluorophosphonate (MAFP)^26^, final concentration: 15 μM, Cat: 70660, Cayman Chemical, USA) or LR blocking antibody (MLuC5^27^, final concentration: 20 μg/ml, Cat: ab3099, Abcam, USA) under concentrations without cytotoxicity were used to stimulate cells for 1 hour, followed by another 1 hour of PEDF (10ng/ml) treatment. Then cells were moved into hypoxia chamber (Stem cell technologies, USA) maintained at low oxygen tension (1% O_2_, 5% CO_2_ and 94% N_2_) for 8 hours. Finally, RNA was isolated for real time RT-PCR.

### M1- and M2-like macrophages preparation

Bone marrow–derived monocytes were collected from the femoral shafts of adult C57 or enhanced enhanced green fluorescent protein (EGFP) male mice, and they were incubated in 10 mL of RPMI 1640 supplemented with GlutaMAX (GIBCO, Invitrogen, Carlsbad, CA, USA) containing 10% FBS. Then, 50 ng/mL of macrophage colony-stimulating factor (M-CSF) (R&D System), a secreted cytokine, was added to the solution. Five days later, some cells were stimulated with 1 μg/mL LPS (Sigma-Aldrich) and 20 ng/mL IFN-γ (R&D System) for 24 hours to induce differentiation into the M1 phenotype. Other cells were stimulated with 20 ng/mL of IL-4 (R&D System) and IL-10 (R&D System) for 24 hours to induce a polarization of the cells into M2 phenotype^46^. The expressions of macrophage markers were determined by real-time RT-PCR or western blot.

### Hypoxia Treatment

Hypoxia-induced M1- and M2-like macrophage polarization was according to the report before. Cells were exposed to hypoxic environment within the hypoxia chamber (Stem cell technologies, USA) maintained at low oxygen tension (1% O_2_, 5% CO_2_ and 94% N_2_). The treatment was initiated by introducing the culture in the hypoxia chamber and replacing the existing culture medium with deoxygenated RPMI 1640/DMEM. The oxygen concentration in the hypoxic chamber and the exposure medium was monitored by using an oxygen indicator (Forma Scientific, Marietta, OH)^47^.

### HUVECs proliferation and tube formation assay

Approximately 4 × 10^3^ cells in 100 μl of DMEM (Invitrogen) containing 0.2% FBS was plated in 96-well plates and incubated under standard culture conditions (37 °C, 5% CO_2_). 24 hours later, the medium was replaced with the supernatant of M0-, M1-, or M2-like macrophages. After 24, 48 or 72 hr of incubation, 10 μl of Cell Counting Kit-8 (CCK-8; Dojindo, Kumamoto, Japan) was added to each well according to the manufacturer’s instructions, and the cells were incubated for another 90 min at 37 °C. Then the absorbance of 450 nm was measured with a microplate reader.

Growth factor-reduced basement membrane matrix (Matrigel; BD Bioscience, San Jose, CA) was added at 150 μl per well into 48-well plates on ice and the plates were placed at 37 °C for 30 min for polymerization. HUVECs (2 × 10^4^) in 100 μl of DMEM containing 0.2% FBS with or without supernatant of M0-, M1- or M2-like macrophages were plated onto the gel surface and incubated at 37 °C for 6 hr. Cell tube formation was examined by phase-contrast microscopy and images of five random fields per well were taken. An endothelial tube length was quantified as described in a previous study^48^.

### Real-time quantitative RT-PCR

Total RNA was prepared using Trizol Reagent (Invitrogen) following to the manufacture’s instructions. RNA was quantified by Thermo NANODROP 2000 spectrophotometer, Total RNA (1 μg) was reverse transcribed using GoScriptTM Reverse Transcription System (Promega) according to the manufacturer’s instructions. Q-PCR were performed on Mx3005 P (Stratagene) using GoTaq qPCR Master Mix (Promega). The following gene expression assays were purchased from Invitrogen: GAPDH: GGTTGTCTCCTGCGATTCA (forward) and TGGTCCAGGGTTTCTTACTCC (reverse), IL-1β: TGCCACCTTTTGACAGTGATG (forward) and AAGGTCCACGGGAAAGACAC (reverse); iNOS: AAGGTCCACGGGAAAGACAC (forward) and ACATTGATCTCCGTGACAGCC (reverse); MCP-1: CTCGGACTGTGATGCCTTAAT (forward) and TAAATGCAAGGTGTGGATCCA (reverse); CD206: GGAATCAAGGGCACAGAGTTA (forward) and ATTGTGGAGCAGATGGAA (reverse); CD163: CAGACTGGTTGGAGGAGAAATC (forward) and TGACTTGTCTCTGGAAGCTG (reverse); VEGF: CACTTCCAGAAACACGACAAAC (forward) and TGGAACCGGCATCTTTATCTC (reverse); VEGFR1: TAGTGTTGTGGGCTCTGTATTC (forward) and AGCTTCCTCAGCACACTATTT (reverse); VEGFR2: AGCAGGATGGCAAAGACTAC (forward) and TACTTCCTCCTCCTCCATACAG (reverse). Quantification of gene expression was determined by the comparative 2^ΔΔ*CT*^ method. The relative expression levels were determined by normalizing expression to glyceraldehyde 3-phosphate dehydrogenase (GAPDH). All the assays were performed in triplicate and repeated at least three times.

### Isolation of mouse retina CD11b^+^ cells

Mouse retinas of P15, P17 and P21 from wild-type (WT) and OIR mice were carefully dissected out and digested in pre-warmed 16.5-U/ml papain solution (Worthington Biochemical) for 30 min with gentle pipetting. Trypsin inhibitor was added to stop digestion. Then the cell digestion suspensions were transferred and passed through cell strainers (BD Falcon) to ensure single-cell suspension, and cells were spun down at 900 rpm (89 g). After gently removing the supernatant, the cell pellet was resuspended with 90 μl MACS buffer (BD Biosciences) and mixed well with 10 μl anti-mouse CD11b magnetic beads (Miltenyi Biotec), incubated at 4 °C for 20 min, washed once, and resuspended in 500 μl MACS buffer. The cell pellet was then loaded on a premoisturized MS column (BD Biosciences) and washed twice. Then the columns were taken off the magnetite and residue cells were flushed out of the column, according to the manufacturer’s protocol. The selected cells were collected and analyzed by flow cytometry. All retina cell samples from different mice were treated as mentioned separately.

### Flow cytometry analysis

The CD11b^+^ cells from the retinas of mice with ROP were resuspended in MACS buffer (BD Biosciences) and incubated with phycoerythrin-conjugated anti-mouse CD11c (eBioscience), fluorescein isothiocyanate-conjugated F4/80 (eBioscience), Alexa Fluor 647-conjugated CD206 (AbD), and the matching control isotype IgGs for 30 min at 4 °C. Then the cells were washed and rinsed again and incubated with secondary antibodies for 30 min at 4 °C. The cells were then washed and resuspended in FACS buffer (BD Biosciences) and analyzed by flow cytometry (FACSCalibur cytometer). M1 macrophages were identified as F4/80^+^/CD11c^+^ and M2 macrophages as F4/80^+^/CD206^+^ cells^49^. Data analyses were performed using FLOWJO software (Tree Star).

### Statistical analysis

All data are present as mean ± SEM. We used two-tailed t tests to determine significances between two groups. We did analyses of multiple groups by one-way or two-way ANOVA with Bonferroni post test of GraphPad prism version 5. For all statistical tests, we considered *P* value < 0.05 to be statistically significant.

## Additional Information

**How to cite this article**: Gao, S. *et al*. PEDF mediates pathological neovascularization by regulating macrophage recruitment and polarization in the mouse model of oxygen-induced retinopathy. *Sci. Rep.* **7**, 42846; doi: 10.1038/srep42846 (2017).

**Publisher's note:** Springer Nature remains neutral with regard to jurisdictional claims in published maps and institutional affiliations.

## Supplementary Material

Supplementary Figures
