# Supplementary material for: PEDF mediates pathological neovascularization by regulating macrophage recruitment and polarization in the mouse model of oxygen-induced retinopathy
Source: Sci Rep. 2017 Feb 17;7:42846. doi: 10.1038/srep42846 (PMC5314378; doi:10.1038/srep42846)
Supplement: Supplementary Figures [file srep42846-s1.pdf]

PEDF mediates pathological neovascularization by regulating macrophage recruitment and polarization in the mouse model of oxygen-induced retinopathy

Sha Gao[1 #], Changwei Li[2 #], Yanji Zhu[1], Yanuo Wang[1], Ailing Sui[1], Yisheng Zhong[1]*, Bing Xie[1]*, Xi Shen[1]*

[1] Department of Ophthalmology, Ruijin Hospital, Shanghai Jiaotong University School of Medicine, 197 Ruijin 2nd Road, Shanghai, 200025, People's Republic of China.

[2] Shanghai Key Laboratory for Prevention and Treatment of Bone and Joint Diseases with Integrated Chinese-Western Medicine, Shanghai Institute of Traumatology and Orthopedics, Ruijin Hospital, Shanghai Jiaotong University School of Medicine, 197 Ruijin 2nd Road, Shanghai, 200025, People's Republic of China.

# These authors contributed equally to this work

* Corresponding authors:

Xi Shen

The Department of Ophthalmology, Ruijin Hospital, Shanghai Jiao Tong University School of Medicine, Shanghai, China; Address: No. 197, Ruijin 2nd Road, Shanghai 200025 China; Zip Code: 200025; Phone: +86 21 64335742; Fax: +86 21 64370045; E-mail: carl_shen2016@126.com

Yisheng Zhong

The Department of Ophthalmology, Ruijin Hospital, Shanghai Jiao Tong University School of Medicine, Shanghai, China; Address: No. 197, Ruijin 2nd Road, Shanghai 200025 China; Zip Code: 200025; Phone: +86 21 64335742; Fax: +86 21 64370045; E-mail: zhongyis2016@126.com

Bing Xie

The Department of Ophthalmology, Ruijin Hospital, Shanghai Jiao Tong University School of Medicine, Shanghai, China; Address: No. 197, Ruijin 2nd Road, Shanghai 200025 China; Zip Code: 200025; Phone: +86 21 64335742; Fax: +86 21 64370045; E-mail: bingxie2016@126.com

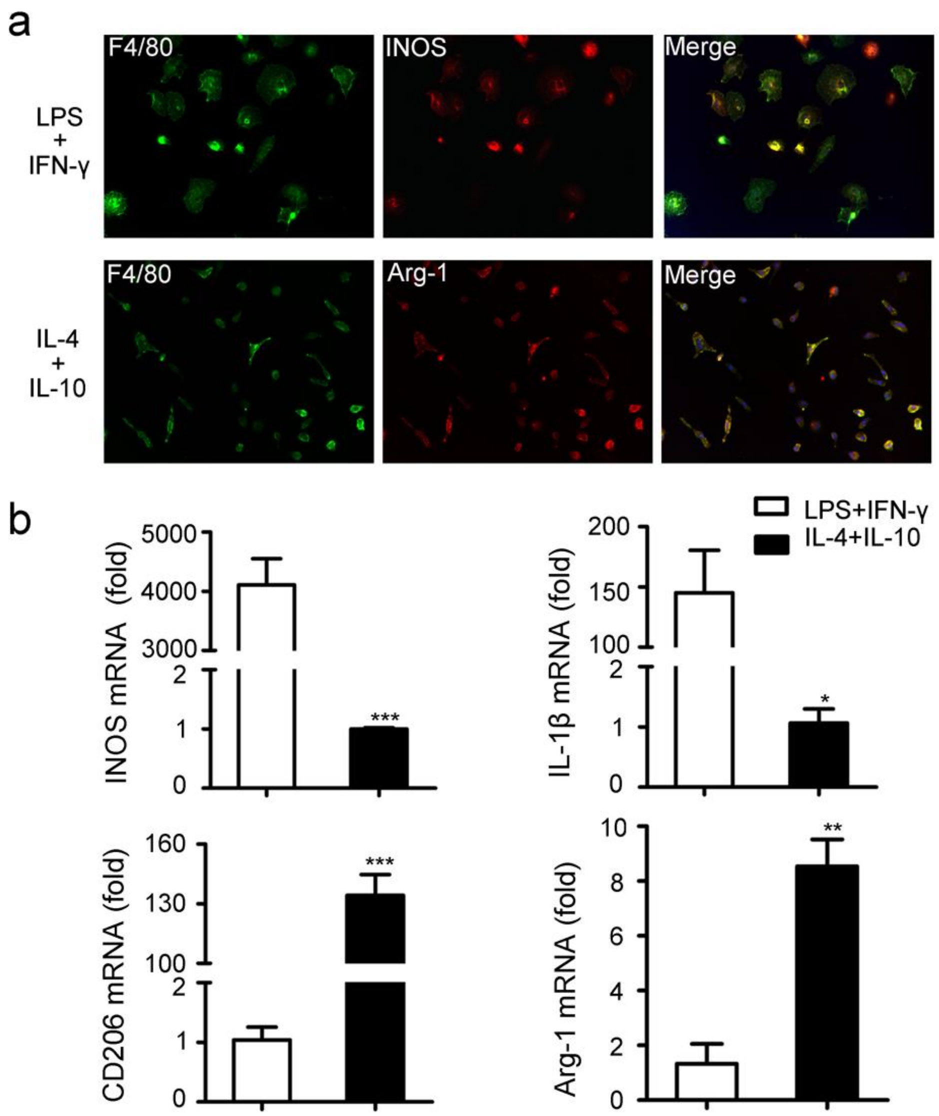

Supplementary figure 1, M1- and M2-like macrophages related genes expression induced by LPS plus INF-γ or IL-4 plus IL-10. (a) Immunofluorescence analysis of F4/80 and iNOS in bone marrow derived macrophages induced by LPS plus INF-γ or IL-4 plus IL-10. (b) Quantitation of iNOS, IL-1β, CD206 and Arg-1 expression in bone marrow derived macrophages induced by LPS plus INF-γ or IL-4 plus IL-10. **P*<0.05, ***P*<0.01, ****P*<0.001. *P* values were analyzed by two-tailed t tests. All data are representative of three independent and are means ± SEM.

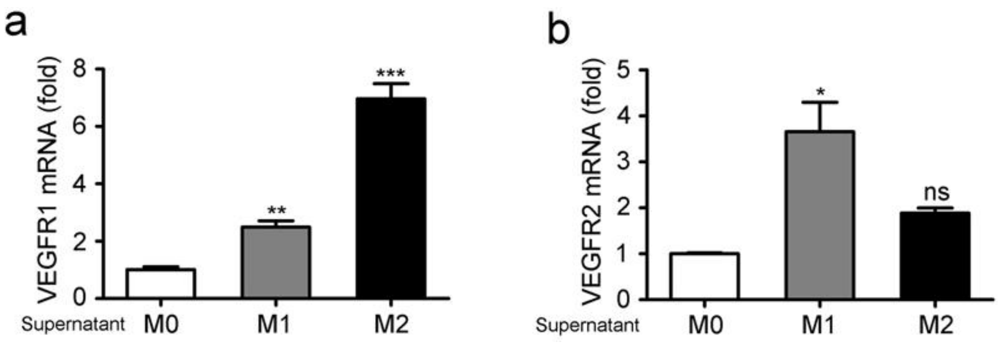

Supplementary figure 2, VEGFR1 and VEGFR2 expression in HUVECs induced by the supernatant of M0-, M1- and M2-like macrophages. (a&b) Quantitation of VEGFR1 (a) and VEGFR2 (b) in human umbilical vein endothelial cells (HUVECs) post 24 hours incubated with the supernatant of M0-, M1-, or M2-like macrophages. *$P<0.05$, **$P<0.01$, ***$P<0.001$. P values were analyzed by one-way ANOVA. All data are representative of three independent experiments and are means ± SEM.
